# Xenobiotics Formed during Food Processing: Their Relation with the Intestinal Microbiota and Colorectal Cancer

**DOI:** 10.3390/ijms20082051

**Published:** 2019-04-25

**Authors:** Alicja M. Nogacka, María Gómez-Martín, Adolfo Suárez, Oscar González-Bernardo, Clara G. de los Reyes-Gavilán, Sonia González

**Affiliations:** 1 Department of Microbiology and Biochemistry of Dairy Products, Instituto de Productos Lácteos de Asturias, Consejo Superior de Investigaciones Científicas (IPLA-CSIC), Paseo Río Linares s/n, Villaviciosa, 33300 Asturias, Spain; alicja.nogacka@ipla.csic.es; 2 Diet, Microbiota and Health Group, Instituto de Investigación Sanitaria del Principado de Asturias (ISPA), Oviedo, 33011 Asturias, Spain; mariagomart@gmail.com (M.G.-M.); adolfo.suarez@hcabuenes.es (A.S.); 3 Department of Functional Biology, University of Oviedo, C/Julian Clavería s/n Oviedo, 33006 Asturias, Spain; 4 Digestive Service, Central University Hospital of Asturias (HUCA), SESPA, Oviedo, 33011 Asturias, Spain; oscargbernardo@hotmail.com

**Keywords:** xenobiotics, heterocyclic amines, aromatic polycyclic hydrocarbons, colorectal cancer, intestinal microbiota, diet, cooking, food processing, genotoxicity, cytotoxicity

## Abstract

The colonic epithelium is exposed to a mixture of compounds through diet, among which some are procarcinogens, whereas others have a protective effect. Therefore, the net impact of these compounds on human health depends on the overall balance between all factors involved. Strong scientific evidence has demonstrated the relationship between nitrosamines (NA), heterocyclic amines (HCAs), and polycyclic aromatic hydrocarbons (PAHs), which are the major genotoxins derived from cooking and food processing, and cancer. The mechanisms of the relationship between dietary toxic xenobiotics and cancer risk are not yet well understood, but it has been suggested that differences in dietary habits affect the colonic environment by increasing or decreasing the exposure to mutagens directly and indirectly through changes in the composition and activity of the gut microbiota. Several changes in the proportions of specific microbial groups have been proposed as risk factors for the development of neoplastic lesions and the enrichment of enterotoxigenic microbial strains in stool. In addition, changes in the gut microbiota composition and activity promoted by diet may modify the faecal genotoxicity/cytotoxicity, which can be associated with a higher or lower risk of developing cancer. Therefore, the interaction between dietary components and intestinal bacteria may be a modifiable factor for the development of colorectal cancer in humans and deserves more attention in the near future.

## 1. Introduction

Both diet and intestinal microbiota are considered to be two major factors that influence colonic health and the incidence of intestinal disorders such as colorectal cancer (CRC) [1]. It is generally accepted that diet acts as an essential factor for health maintenance. However, after several decades of research, the specific dietary compounds implicated in this protective effect have not yet been determined, which presents the opportunity to define an optimal diet.

This review discusses the impact of diet on CRC, the generation of xenobiotics, the interactions between these xenobiotics and gut microbiota, and identifying the factors that contribute to a balance of the factors necessary for a healthy gut. We then discuss the perspectives that can be used to guide our understanding of the contributions of diet and microbiota in protection against CRC.

### 1.1. Impact of Diet on Colorectal Cancer

Since the beginning of agriculture and animal husbandry approximately 10,000 years ago, humans have been exposed to profound changes both in diet and lifestyle that generally have taken place too rapidly to allow the fixation of genetic adaptations in the population [2]. This has prompted many authors to suggest that the current increase in the prevalence of the so-called “diseases of civilization” may be the result of discordance between the human Palaeolithic gastrointestinal system and modern diets.

Considering scientific evidence regarding the link between diet and health for the most generalized patterns worldwide, Western diets (WDs) are characterized by a high consumption of fatty and sugary foods, salt, sauces, meat and meat products, and processed foods [3]. Strong scientific evidence has indicated a protective role of the Mediterranean diet (MD) against the development of some high-prevalence and non-communicable pathologies in developed countries such as CRC [4,5], while adherence to a Westernized dietary pattern has been recognized as a potential risk factor.

A meta-analysis of thirteen prospective cohort studies concluded that a high-fat diet did not increase the risk of CRC [6], and no reduction in the risk of this disease was found with a low-fat diet after an eight-year follow-up in a randomized clinical trial [7]. Some authors have also evaluated the effect of fruit and vegetable consumption on colon cancer [8]. This risk of CRC increased when the consumption of these two food groups was below 300 g/day [8]. To the contrary, the results from the European Prospective Investigation into Cancer and Nutrition study (EPIC), one of the largest cohort studies in the world, did not support a significant inverse association between the consumption of fruits and vegetables and the occurrence of CRC, suggesting that there was little benefit of increasing the consumption of fruits and vegetables in comparison to the protection associated with an overall balanced diet [9]. The most recent revision of the Continuous Update Project from the World Cancer Research Fund International demonstrated that consumption of 90 g/day of whole grain is associated with a decrease in the risk of colon cancer, mainly attributable to the fibre content of whole grains [8]. Among the different components included within the concept of the WD, meat and meat products have accumulated the strongest scientific evidence in the context of CRC [10,11,12] and have been classified recently by the International Agency for Research on Cancer (IARC) as likely carcinogenic and carcinogenic foods, respectively [13]. A recent systematic review and meta-analysis of cohort studies have associated red meat with a significant increase in the risk of CRC (relative risk (RR) for 100 g/day of increase: 1.22; confidence interval (CI) 95%: 1.06–1.39). Nevertheless, this association was not observed for rectal cancer (RR: 1.13; CI 95%: 0.94–1.34). In addition, whereas the intake of processed meat has been shown to significantly increase the risk of colon cancer (RR: 1.23; CI 95%: 1.11–1.35), this association was marginally significant for rectal cancer (RR: 1.08; CI 95%: 1.00–1.18) [13,14]. It has been estimated that the risk of CRC increases 17% for every 100 g of red meat consumed per day [15], a risk that would be augmented in the case of chemically treated red meats [15,16]. This information is of high importance considering that in most developed countries, as is the case in Spain, the consumption of processed meat has increased in recent years, reaching 8 kg/capita/year in 2017 [17], while the intake of fruits and vegetables during the same period of time has gradually decreased [17]. The different mechanisms by which dietary patterns may be related to health are diverse in nature. In the literature, the net effect of the WD on CRC has been mostly related to the overall balance between low contents of antioxidants, fibre and polyunsaturated fatty acids and a high proportion of foods with a low-density high glycaemic index and rich in animal fats. In addition, in recent decades, major emphasis has been placed on the link between the ingestion of cooked and processed foods and the risk of colon cancer [18].

### 1.2. Intestinal Microbiota and Human Health

The gastrointestinal tract is inhabited by a dense microbial community known as the ‘microbiota’ that is composed of viruses and members of the three domains of life: bacteria, archaea and eukarya [19]. Metagenomic studies estimate that the total bacteria in our body exceeds approximately 10 times the number of nucleated eukaryotic cells [20], harbouring a genetic potential 100-fold larger than that of the whole human genome [21]. The intestinal bacterial population is mainly composed of members belonging to just two phyla, Bacteroidetes and Firmicutes, both constituting approximately 80–90% of the microorganisms in this habitat. Other subdominant microorganisms, in decreasing order of abundance (less than 10% of total intestinal bacteria), are members of the phyla Actinobacteria, Proteobacteria and Verrucomicrobia, respectively [22]. The intestinal microbiota carry out crucial functions that are beneficial to the host [23] and that could be mainly grouped as metabolic-degradation of non-digestible carbon sources and production of different metabolites such as vitamins and short chain fatty acids (SCFAs), protective-inhibition of pathogen adhesion to intestinal surfaces and trophic-maintenance of the intestinal epithelium integrity and functionality [24].

Intestinal microbial communities vary greatly among individuals, and it is difficult to define a ‘healthy microbiota’. It has been proposed that distinct types of gut microbial communities (‘enterotypes’), driven by diet and defined by their bacterial composition, are mainly characterized by relatively higher levels of a single bacterial genus: *Prevotella*, *Ruminococcus* or *Bacteroides* [22]. However, the vast interindividual variability of the microbiota demonstrated by large-scale metagenomic studies indicates that these differences are distributed in the population as continuous gradients of dominant taxa rather than discrete defined clusters [25,26,27]. Considering the concept of a healthy microbiota, despite this interindividual taxonomic variability, the functions of the gut microbiota remain relatively stable among individuals as there is a functional redundancy among the diverse members of this microbial community. Thus, a “core microbiome” constituted by specific microbial gene family combinations, metabolic modules, and regulatory pathways collectively promoting a stable host-associated ecology, could be ideally defined [28]. Gender, age, body mass, ethnicity, geographic location, and immune status [28] are intrinsic factors that influence the concept of a “healthy microbiota”. However, it must be noted that the influence of the microbiota on other factors is usually not considered, such as the intestinal transit time or previous drug consumption [28]. An imbalance in the composition and functionality of the microbiota occurring in several diseases is known as dysbiosis. The link between disease and microbiota has been repeatedly replicated in experiments with faecal transplantation in mice and the reproduction of the initial altered phenotype [29,30]. However, it is still challenging to determine whether changes in the microbiota are the cause or consequence of the disease [31]. One of the most common events occurring in dysbiosis states is a decrease in intestinal bacterial richness, frequently accompanied by variations in the relative abundance of some microbial genes and functions that differ among pathologies, as has been described in obesity [32], inflammatory bowel disease [33], autism [34], and CRC [35], among others. Specifically, the dysbiosis associated with CRC is generally characterized by an increase in the prevalence of pathogenic or pathogen-associated microorganisms from genera *Fusobacterium*, *Porphyromonas*, *Peptostreptococcus*, *Parvimonas* and *Enterobacter* and by a depletion of gram-positive fibre-fermenting Clostridia [35,36]. Furthermore, the consumption of chemically and thermally processed foods and the adherence to a WD have been shown to drive specific changes in gut microbiota composition and activity [37,38] towards the production of metabolites with potential carcinogenic effects [39]. Thus, a comprehensive understanding of how these compounds derived from food processing can interact with the microbiota and microbiome is necessary to determine their true impact on overall gastrointestinal health [40].

## 2. Food Processing and Xenobiotics

**Figure 1:**
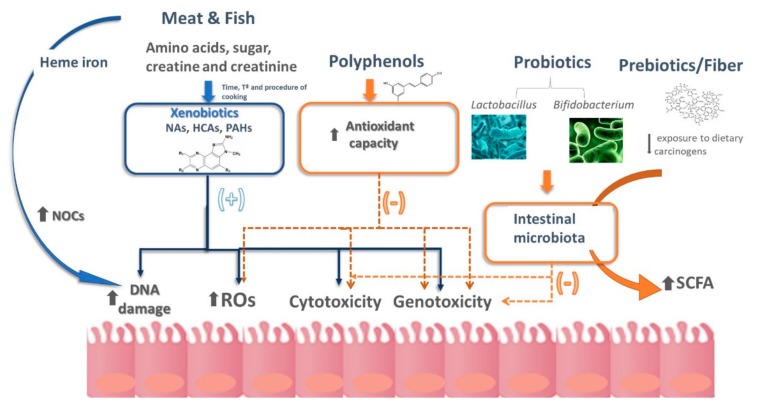
Schematic representation of the different hypotheses currently available that contribute to explaining the relationship between diet and colorectal cancer. HCAs, heterocyclic amines; NAs, nitrosamines; NOCs, *N*-nitroso compounds; PAHs, polycyclic aromatic hydrocarbons; ROS, reactive oxygen species; SCFA, short chain fatty acids. More details are explained in the text.

Although the exact mechanism by which meat is related to cancer is unknown, several authors have postulated that the thermal formation of different carcinogens during cooking, such as heterocyclic amines (HCAs) and polycyclic aromatic hydrocarbons (PAHs), the addition of *N*-nitroso compounds (NOCs) to cured meats, the endogenous NOC formation from haem iron and the generation of lipid and protein oxidation products, are included within the possible mechanisms underlying this association (Figure 1) [41].

In this regard, haem iron, in addition to being related to the production of NOCs at the intestinal level, has been associated with the generation of aldehydes with cytotoxic and genotoxic properties [42]. Additionally, meat processing involves the addition of nitrites, salt, smoke and the application of different grades of temperature depending on the cooking method [11], all of which are related to an increased risk of colon cancer. From xenobiotics, HCAs have accumulated the strongest scientific evidence as cancer risk factors in epidemiological and interventional studies (Table 1) and were classified by the IARC as potential carcinogens. In recent years, more than twenty-five HCAs have been identified in regular food products [43], formed from creatinine, creatine, hexoses, amino acids and some dipeptides, which are present mainly in the muscle of meats and fish [44,45].

HCAs can be classified into two large groups according to their molecular structures and metabolic pathways: aminocarbolines (ACs), or pyrolytic amines, and aminoimidazoazarenes (AIAs), or thermal amines. ACs are formed by the pyrolysis of proteins at temperatures above 300 °C, while AIAs are generated by applying temperatures from 100 to 300 °C to dietary sources of sugars, amino acids and creatinine [49]. As the mutagenic activity of HCAs increases with temperature [50] and with the browning degree of cooked food, cooking methods such as frying, grilling or roasting lead to the formation of higher amounts of HCAs than boiling, steaming or braising [51]. In this regard, data from the EPIC revealed the existence of a large variation in the intake of these foodstuffs and in the cooking methods among European countries. The Netherlands has been found to be the population with the highest intake of red meat prepared at high temperature (mean intake of 39.4 g/day and 59.7 g/day for women and men, respectively [52].

PAHs are formed in a large variety of foods, including oils, grains and vegetables, after applying a heat treatment for cooking (frying, baking, grilling, etc.) or processing. Among the different types of PAHs classified by the IARC, benzo(a)pyrene (BaP) has been classified as carcinogenic to humans [53]. Nevertheless, given the ubiquity of PAHs in food and their presence as contaminants, it is very difficult to assess to what extent the amount ingested from food may contribute to cancer development. PAHs [54] can be formed by pyrolysis of organic matter at high temperatures, by direct contact of lipid droplets with a heat source, by the smoke produced during cooking, or by the incomplete combustion of coal or wood in barbecues or grills [55,56,57]. The maximum levels of PAHs have been found in smoked foods and grilled meats [57].

As with many other dietary components, the impact of xenobiotics related to food processing on health depends on the dose of intake and the frequency of exposure to the toxic agent/s. In this regard, some authors have highlighted that chronic exposure to contaminants may progressively induce a low-grade inflammatory status in the host, partly mediated by the aryl hydrocarbon receptor, a cytosolic transcription factor activated by different hydrophobic chemicals [58] and present in different mammalian cells. With considerable variation among countries, the amount of HCAs consumed mainly depends on the cooking method, temperature, the meat or fish itself and the nutritional composition of the foodstuffs [59,60]. However, these factors are very difficult to assess accurately through dietary questionnaires for several reasons. First, cooking methods are highly variable over time. Second, there are no standardized tools currently available, such as photographs of scales, to quantify the degree of browning in foods, so there is high variability between studies. Third, the interaction between the different components of the diet is too difficult to determine long-term. In addition, after the intake of red meat, other carcinogenic compounds associated with many processed meats, such as NOCs, can be formed endogenously [59] by the intestinal microbiota and can be activated to act as carcinogens/mutagens [61].

## 3. Effect of Food Processing-Borne Xenobiotics on the Gut Microbiota

The human colon is exposed to multiple compounds of dietary origin, as well as those resulting from digestion, intestinal microbial metabolism, and host excretory processes. Intestinal microbiota is known to produce faecal metabolites, with genotoxic and mutagenic potential, some of which have been compiled on Table 2.

Cytotoxicity is the capability of certain substances to cause cell injury, with deleterious effects on metabolism, structure and/or viability of cells. Genotoxicity is the capability to induce damage to cellular genetic material, altering the DNA sequence or modifying its structure; more specifically, mutagenicity refers to the capacity of some genotoxic agents to produce alterations (mutations) in the DNA sequence. Some potential faecal mutagens can be produced by the intestinal microbiota, including microbial genotoxins [62]. Other compounds are formed endogenously from dietary constituents, such as nitrates, dietary amines and cholesterol, or are synthesized from precursors originating from human metabolism, such as NOCs, fecapentaenes, long chain fatty acids, and secondary bile acids generated by the metabolism of intestinal bacteria [63]. Table 2 summarizes the cytotoxic and genotoxic mechanisms of endogenous molecules and compounds generated by the intestinal bacteria that could be involved in CRC. A group of toxic substances are from exogenous origins and include mycotoxins, plant glycosides, some food additives and notably, two groups of xenobiotics discussed in this review that are formed by pyrolysis during food cooking and processing: HCA and PAH.

Studies of faecal genotoxicity and mutagenicity related to dietary habits have revealed different levels of toxicity associated with different dietary patterns [85], as well as an association between high faecal genotoxicity and an augmented risk of CRC [63]. Although some authors have suggested the possibility of using faecal genotoxicity as a preventive and early marker for the risk of CRC [86,87], more studies are needed to support this proposal. The relationship between cytotoxicity and intestinal disease is currently less clear. Moreover, it is necessary to elucidate the causal role of the different genotoxic and cytotoxic compounds on CRC and the influence of dietary patterns, intestinal microbiota, host physiology and lifestyle on the resulting toxicity in the intestinal environment. In this sense, some studies indicate that the presence of co-mutagenic, inhibitory or potentiating factors could modify the toxicity of genotoxic and cytotoxic compounds and hence the resulting intestinal toxicity [87,88,89].

### 3.1. Impact of Xenobiotics on Gut Microbiota

The human gut microbiota interacts with food xenobiotics in dual ways: xenobiotics influence the microbiota, and in turn, the microbiota can also metabolize and transform xenobiotics, altering their toxicity. Few studies are currently available that evaluate the impact of food xenobiotics on the gut microbiota and the consequences on the host immune system and metabolism. Ribière et al. (2016) found that oral exposure to benzo(a)pyrene led to moderate inflammation in ileal and colonic mucosa [90] and induced changes in the gut microbiota composition, without affecting the alpha-diversity index, in a murine model. Among dominant intestinal taxa, bacterial families such as *Bacteroidaceae*, *Porphyromonadaceae* and *Paraprevotellaceae* showed a significant increase in their relative abundance, whereas *Lactobacillaceae* and *Verrucomicrobiaceae* (only represented by *Akkermansia muciniphila*) decreased; in contrast, among the less abundant microorganisms, the *Actinobacteria* class (mainly represented by the genus *Bifidobacterium*) and some members of the *Coriobacteriaceae*, *Rikenellaceae*, and *Desulfovibrionaceae* families displayed increased abundance after mice were exposed to benzo(a)pyrene. Interestingly, Defois et al., using in vitro faecal models, demonstrated important changes in the metabolome and transcriptome of the human gut microbiota upon exposure to a variety of food contaminants without affecting the structural composition of the microbial community thus highlighting important changes in microbial metabolic activity [91]. Changes in the volatolome affected sulphur, phenolic and ester compounds. The transcriptome revealed an increase in lipid metabolism processes, cell wall/plasma membrane/periplasmic space and DNA repair and replication systems, whereas the transcription of genes related to glycolysis/gluconeogenesis and bacterial chemotaxis towards simple carbohydrates as well as ribosome, translation and nucleic acid binding was downregulated [91,92].

### 3.2. Impact of the Gut Microbiota on the Toxicity of Xenobiotics

The intestinal microbiota has the capacity to modify the toxicity of food xenobiotics by direct microbial interference with these compounds and/or by modulating host-microbial interactions. First, some lactic acid bacteria (LAB) and other microorganisms present in the human gut can directly bind or metabolize diet-derived HCAs or other xenobiotics [93,94,95,96], contributing either to the sequestration and excretion of these compounds in faeces or to their transformation into less toxic compounds, which potentially helps to prevent DNA damage and generation and progression of pre-neoplastic lesions [97]. The gut microbiota can also metabolize xenobiotics transforming them into chemically derived molecules with enhanced mutagenic activity [98]. Thus, additional studies are necessary to elucidate the range of bacteria capable of carrying out each of these transformations of different xenobiotics originating during food processing, the metabolic effects of such biotransformation on the microbiota and host, and the relevance of these transformations to intestinal disease and carcinogenesis.

The modification of the toxicity of xenobiotics can also occur via host-microbiota interactions. The most known of these interactions is the exacerbation of the toxicity of xenobiotics through enterohepatic cycling. Xenobiotics are often conjugated to glucuronic acid in the liver (one of the pathways of phase II detoxification in the human body), stored in the gallbladder, and released into the intestine with bile during digestion. When the conjugated xenobiotic enters the intestine, the microbial β-glucuronidases can cleave the deactivated glucuronidated molecule and release the unconjugated xenobiotic, turning it back again into a toxic molecule [99]. The de-glucuronidation of xenobiotics by microbial β-glucuronidases, such as those found in many enterobacteria and in some microorganisms from the *Clostridium* and *Bacteroides* groups [98], is a phenomenon already demonstrated to occur with some HCAs [97,100], but it remains unknown whether it could be a general detoxification mechanism that also affects other HCAs and PAHs. Another possible way of increasing the toxicity of xenobiotics in the gut is the alteration of host gene expression by the microbiota. Cytochrome P450 comprises different hepatic enzymes that participate in phase I of detoxification. One of these enzymes, CYP1A1 (aryl-4 monooxygenase), has been linked to the intestinal detoxification of benzo(a)pyrene depending on TLR2, a host cell membrane receptor triggered by bacterial lipoproteins and other cell wall components; TLR2-deficient mice had reduced ability to clear benzo(a)pyrene and developed colon polyps after dietary supplementation with this compound [101]. The results of the study of Do and colleagues suggest the interesting possibility that the gut microbiota could modulate the host xenobiotic metabolism through TLR2 signalling [97].

Conventional tests available for the study of genotoxicity, mutagenicity and cytotoxicity of faecal waters are mostly based on those developed to routinely characterize potential hazards of chemicals, as indicated in the Organization for Economic Cooperation and Development (OECD) Health Effects Test Guidelines [102,103]. Some tests are available for determining genotoxicity in vitro, in vivo and/or ex vivo; the comet test is the most commonly used, but others, such as the micronucleus assay (MN), sister chromatid exchange assay (SCE) or the SOS chromotest are also used [104]. The Ames test is by far the most extensively applied to assess in vitro mutagenicity and is based on the capacity to cause reverse mutations in defective genes for essential amino acids (such as histidine or tryptophan) in auxotrophic strains of *Salmonella* Typhimurium and *Escherichia coli*.

Intestinal cytotoxicity could be evaluated in adenocarcinoma cell lines, generally Caco-2 and HT-29. Some assays determine the effect of exposure to toxic agents on cell proliferation capacity [101]. Cytotoxicity can also be evaluated by determining mitochondrial function or membrane integrity [105]. Among the most commonly used are the dye exclusion test and the MTT assay [105]. Real-time electronic sensing (xCELLigence system; Roche, Basel, Switzerland) monitors variations in the impedance of cultures of carcinogenic cell lines at the proliferation or confluence states using gold microelectronic sensor arrays [106,107].

In spite of the classical methods available, the interpretation of faecal mutagenicity/genotoxicity related to intestinal disease and CRC risk is hindered by the limitation of analytical power when applied to the study of human samples, by the inaccuracies and errors of the experimental techniques used, and because the in vitro and in vivo tests may not adequately reflect what happens in the complex human intestinal ecosystem. Recent advances in the culture of pluripotent stem cells and primary tissues have made possible the culture, differentiation and self-assembly of many cell types and structures from true organs in three dimensions to form the so-called “intestinal organoids”. These organoids could allow the construction of human or animal-specific models for the study of intestinal toxicity [108]. This cutting-edge scientific research tool still presents some limitations, as with any technology. The most remarkable is non-vascularization due to limitations in nutrient supply in culture media, the limited presence of stromal cells, including those from the immune system, and the lack of reliable means to synchronize the shape, size and viability of organoids in culture [109]. Beyond the limitations of the analytical and predictive power of the techniques available to determine levels of intestinal toxicity and the necessary improvement of their accuracy, the emerging omics technologies (metagenomics, RNA-seq, metabolomics, and culturomics) are complementary and powerful skills that could help to shed light on the specific metabolic cell routes altered by toxic compounds, contributing to the exploration of new biomarkers for genotoxicity assessment related to intestinal disease and cancer risk.

## 4. Creating a Balance between Xenobiotics and a Healthy Gut

As mentioned previously in this review, some gastrointestinal microbes are able to generate toxic compounds themselves and/or to convert pre-carcinogens into carcinogens, whereas some commensal bacteria, probiotics, prebiotics and dietary compounds can act as inhibitors or attenuators of the genotoxicity of xenobiotics. Therefore, the resulting damage at the intestinal level will depend on the interaction among these three factors: the intake of potentially toxic xenobiotic formed during food cooking and processing, the global subject´s diet and the host intestinal microbiota profile. The optimum diet for the prevention of CRC has not yet been defined, but it is well accepted that diets rich in fat and meat and poor in vegetables and fibre tend to increase faecal genotoxicity and the risk of CRC. Apart from this traditional approach, the existence of an interactive effect between phenolic antioxidants and HCA-induced carcinogenesis was proposed twenty years ago based on the capacity of phenolics to inhibit the metabolic activation of HCAs [18] and influence cell proliferation, DNA repair enzymes and apoptosis [110] in the host, among other mechanisms [18]. Since then, diverse phytochemicals from different vegetables and vegetable drinks, such as flavonoids from tea [111,112], citrus [113], fruits [114,115], or aromatic species [116], have been reported to inhibit the production of HCAs [117].

## 5. Future Perspectives

It is clear that wider cohort studies are needed to obtain a clear perspective of the long-term impact that the continued intake of xenobiotics formed during food processing has on CRC, considering the tendency of increased consumption of processed foods and the progressive ageing of the population in industrialized countries, which augments the time of exposure. At the same time, some food ingredients may contribute to minimizing the risk associated with this chronic exposure to xenobiotics. The following sections will analyse both perspectives facing the impact of xenobiotics on CRC and highlight the need for more research in the field of xenobiotics derived from food processing.

### 5.1. Probiotics and Prebiotics to Counteract the Effect of Pro-Carcinogenic Compounds

Probiotics are defined as live microorganisms that, when administered in adequate amounts, confer a health benefit to the host [118]. In the recent definition of prebiotics proposed by Bindels et al. in 2015 [119], these are considered “nondigestible compounds that, through their metabolization by microorganisms in the gut, modulate composition and/or activity of the gut microbiota and thus confer a beneficial physiological effect on the host”. This definition includes not only nondigestible oligosaccharides but also most dietary fibres. Both probiotics and prebiotics are included in functional food products and supplements or are part of natural foods and have been the subject of extensive research in recent years. There is currently interest in improving our understanding of the interaction mechanisms between probiotics and prebiotics with intestinal procarcinogenic compounds from diet.

Several probiotic strains from the *Bifidobacterium* and *Lactobacillus* genera have shown beneficial effects in murine models of colon cancer by promoting the inhibition of aberrant crypt foci formation (early predictors of tumour incidence), suppressing tumour growth, inducing cell apoptosis, or ameliorating inflammation [120,121,122]. These events were associated in most cases with a reduction in intestinal genotoxicity and cytotoxicity [89]. Direct physical binding is the most common method for the removal of carcinogenic compounds by LAB and probiotics (mainly from the *Lactobacillus* and *Bifidobacterium* genera) [123]; generally, peptidoglycan is the cell molecule linked to this effect, although the exopolysaccharides produced by some microorganisms can also act as binders of mutagens [124]. Probiotic bacteria tend to maintain high viability in vitro in the presence of food xenobiotics and bind to these compounds; this could facilitate the removal of aggregated particles from the human body, although this mechanism has not yet been specifically proven in the human gut [125]. Another way by which LAB and probiotics can reduce the genotoxicity/mutagenicity is the metabolic modification/inactivation of procarcinogenic molecules [126,127,128]. The antioxidant capacity of some LAB and probiotics may reduce the cytotoxic effect and oxidative damage of some procarcinogens [129]. Probiotics can also contribute to decreasing toxicity through the modulation of the intestinal microbiota composition and its metabolic activity by reducing the microbial production of enzymes involved in the re-activation in the gut of dietary carcinogens and other toxic compounds, such as β-glucuronidases [130]. The interaction with the host immune system, stimulation of host enzymes (such as glutathione transferases, reductases and peroxidases linked to phase I of detoxification primarily) related to the inactivation of carcinogens, modulation of the host’s immune response and regulation of apoptosis are also mechanisms by which LAB ingested with foods or present in the intestine can modify the effect of procarcinogens in the human body [125].

Epidemiological evidence links dietary fibre with colonic health [131]. The mechanisms by which dietary fibres and prebiotics exert a protective effect against cancer can be diverse, and most of them are likely mediated by the intestinal microbiota. Among the physical mechanisms, the increase in bulking associated with high fibre consumption potentiates sequestration of carcinogens by fibre, favouring the elimination of toxic compounds and reducing host exposure. Prebiotics may also exert a protective effect through changes in the composition and activity of the intestinal microbiota by enhancing the production of host xenobiotic-deactivating enzymes, and/or reducing the production, by the intestinal bacteria, of procarcinogenic-producing/activating enzymes (i.e., β-glucuronidases). The protective effect could also be exerted by the activation of xenobiotic-modifying enzymes of the host through the intestinal microbiota, in which at least two mechanisms could be potentially involved: (1) the microbial production of SCFAs, mainly butyrate, by colonic fermentation of prebiotics, can activate the hepatic phase II enzymes that detoxify toxic compounds by conjugation, and (2) the release of phenolic compounds from vegetable fibres (including polysaccharides, lignins and oligosaccharides) in the human colon can act as modulators of the production of xenobiotic-deactivating enzymes by the host [132]. In addition, prebiotics can also modify the host immune response against procarcinogens and can also alter the gene expression of intestinal host tissues through microbiota-host relationships. In this way, the modification by prebiotics of the intestinal microbiota can promote the activation of some enzymatic glycosidic activities in colonocytes or can modify local or systemic responses of the host immune system by enhancing the production of proinflamatory/regulatory cytokines and/or immunoglogulins [132,133].

Despite the attractiveness of probiotics and prebiotics as food agents for CRC prevention, the efficacy of probiotics in maintaining colonic health or in preventing colon cancer needs to be well established in controlled clinical trials. More evidence exists for the link between dietary fibre/prebiotics and colonic health, but most of the mechanisms of action remain speculative and need to be experimentally proven.

### 5.2. Longitudinal Studies on Long Term Impact of Xenobiotics Derived from Food Processing

More longitudinal studies are needed to reach a consensus on the effect of xenobiotics on health since the net impact of these compounds will depend on the amount ingested as well as the frequency and time of exposure. With this aim in mind, it is necessary to prioritize the development of standardized questionnaires for the collection of data on dietary intake suitable for this purpose, with a limited number of items but allowing quantification in foods the degree of cooking, temperature applied, and other possible variables. In fact, the adequate assessment of the intake of xenobiotics formed while cooking food is a very difficult task since it is necessary for the subject surveyed to remember details over time, such as temperature of cooking, method of preparation, and degree of toasting. These parameters are subjected to high daily variation, and the information is very difficult to retrieve from some population groups, as the people (children, elderly, workers, hospitalized patients, etc.) that usually eat outside home. In this sense, taking advantage of the rapid progress and widespread use of new technologies, the development of computer applications, including photos or validated models, that are easy to use by the people interviewed, could greatly facilitate this task in the near future. Although the conversion/extrapolation of the intake of cooked foods to particular toxic compounds can be done through composition tables, such as those developed by the EPIC group [134], the use of biological markers, representative of the actual intake of the subject, could be of great relevance to validate and complete the reported information. The interpretation of the impact of food-associated xenobiotics on health should be made with caution, taking into account the global diet of the subject and other factors involved in CRC development. The setup of the most adequate methodology to analyse the cytotoxicity or genotoxicity of the diet could allow the exploration of the existence of potential synergisms or antagonisms between the different components assessed. This finding would be very useful when establishing nutritional strategies for health promotion or for the development of functional foods, incorporating specific dietary components capable of counteracting the potential carcinogenic effect of xenobiotics associated with food processing.

## 6. Conclusions

In the last decade, major emphasis has been placed on the link between the ingestion of thermally processed foods and the risk of CRC. Xenobiotics with potential carcinogenic activity can be generated from meat and fish components through some cooking procedures with high temperatures. These compounds include mainly NOCs, PAHs and HCAs. HCAs have accumulated the strongest scientific evidence as a CRC risk factor in epidemiological and interventional studies. The presence of xenobiotics from food processing contributes to an increase in local genotoxicity, which has been related to an increase in CRC risk. The intestinal microbiota can modify the toxicity of dietary xenobiotics by direct microbial interference with these compounds and/or by modulating host-microbiota interactions through diverse mechanisms. However, diet and dietary components could interact either with the intestinal microbiota or with xenobiotics, contributing to altered gut toxicity. Therefore, the interaction between dietary components and intestinal microbiota could be considered a modifiable factor for the development of CRC in humans. Thus, more studies are needed to determine the long-term impact of xenobiotics derived from food processing on CRC. Specific nutrients, probiotics and prebiotics/fibres have the capacity to reduce intestinal toxicity; their mechanisms of action and efficacy should be carefully evaluated for use as food agents for CRC prevention.

## Figures and Tables

**Table 1:** Observational studies in recent years associating heterocyclic aromatic amines and polycyclic aromatic hydrocarbons with colorectal cancer.

Year	No. Subjects	Analytical Category	Source	Dose	Pathology	Ref.
2018	407,270	*HCAs*	Red meat	n.a ^a^	MeIQx and DiMeIQx association with all anatomical subsites of colorectal cancer. PhIP associations with total colorectal and colon cancers. Not evidenced an association between ingested B(a)P and CRC	[46]
		MeIQx				
		DiMeIQx				
		PhIP				
		*PAHs*				
		B(a)P				
2018	76,657	*HCAs*	Red meat	50 ng/day	Association of HCAs, B(a)P, and mutagenicity index with the risk of colorectal adenomas	[47]
		MeIQx		n.a ^a^		
		DiMeIQx		40 ng/day		
		PhIP		n.a ^a^		
		*PAHs*				
		B(a)P				
2013	total 3707: 1062 cases and 1645 controls	*HCAs*	Red meat	n.a ^a^	Colon cancer	[48]
		MeIQx				
		DiMeIQ				
		PhIP				

^a^ Not available.

**Table 2:** Cytotoxic/genotoxic mechanisms of endogenous molecules and compounds generated by intestinal bacteria that could be involved in CRC. Direct mechanisms refer to those that promote genotoxic and/or cytotoxic action directly. Indirect mechanisms are those that cause damage at different levels, from which a cytotoxic and/or genotoxic action is derived.

Main Mechanism		Molecules/Compounds Involved	Microbial Group	Experimental Approach Used for Study	Mode of Action	Ref.
**Direct mechanisms**	**Genotoxins**	Typhoid toxin	*Salmonella enterica* serovar Typhi	In vitro and animal models	DNAse activity; induction of symptoms characteristic of typhoid fever	[64]
		Cytolethal distending toxin	Proteobacteria	Cell lines and primary cell and mouse models of chronic infections	DNase activity; Proinflamation and carcinogenic potential	[65,66,67]
		Colibactin	*Escherichia coli* group B	Eukaryotic cells	DNA double-strand breaks	[68]
				Epidemiological and animal model	DNA double-strand breaks in vitro and in vivo; enhanced tumour growth by senescence	[69,70]
	**Alteration of host cellular cycle**	Cytotoxin-associated gene A Vacuolating cytotoxin A	*Helicobacter pylori*	Molecular, experimental and epidemiological	DNA damage; Increases IL-8; produces reactive oxygen species (ROS) and nitric oxide; increases concentrations of cyclo-oxygenase 2; decreases apoptosis; and increases cell proliferation	[66,71]
		Enterotoxin	*Bacteroides fragilis*	In vitro and epidemiological	DNA damage; high levels of ROS; Diarrheal disease, associated with colorectal cancer	[62,72]
		Adhesin A	*Fusobacterium nucleatum*	In vitro and epidemiological	Activation of β catenin pathway	[66,73]
		ExoS exotoxin	*Pseudomonas aeruginosa*	In vitro, experimental and epidemiological	Activation of pathways with final mechanism leading to DNA damage; unknown mechanisms in cancer generation	[62,66]
		Cysteine protease-like	*Shigella flexneri*	In vitro and epidemiological	Potassium outflow conducting to ROS production; induce degradation of p53; DNA damage; dysentery	[62,66]
		Avirulence protein A	*Salmonella enterica*	In vitro and mouse model of inflammation-associated cancer	Target β-catenin pathway; colonic tumorigenesis and tumour progression	[66]
		Cytotoxic necrotising factor	*Escherichia coli*	In vitro and animal models	Activates Rho GTPase; modifies cytoskeleton; triggers G1-S transition; downregulate mismatch repair genes; the role of CNF in infections in not clear	[71,74]
		Cycle-inhibiting factor		In vitro	Inhibition of mitosis	[75]
		Secondary bile acids	Anaerobic bacteria with 7-α dehydroxylation activity of primary bile acids	In vitro colon cells and animal models	Changes in physicochemical membrane properties; Apoptosis and genomic damage by ROS; Deoxycholic acid is carcinogenic at high doses and long-term treatment in animal models	[76]
**Indirect mechanisms**	**Oxidative stress**	Reactive oxygen species	*Peptostreptococcus anaerobius*	In vivo, in vitro and epidemiological	Increase of human colon tumour tissues and adenomas; these bacteria increase colon dysplasia in a mouse model of CRC by induction of ROS levels, which promotes cholesterol synthesis and cell proliferation.	[77]
			*Enterococcus faecalis*	In vitro and in vivo models, epidemiological	Induction of ROS, activation of macrophages; promotion of tumorigenesis	[66,78]
			Faecal matrix	In vitro	Unknown reducing agent	[79]
	**Formation of H_2_S**	H_2_S	Sulfate-reducing bacteria	Epidemiological and in vitro models	Promotes instability or cumulative mutations in a predisposed genetic background	[80]
	**Inflammation**	Wall-extracted antigen	*Streptococcus bovis*	Epidemiological and molecular	Activation of cyclo-oxygenase 2, interleukin 8 production, and cell proliferation	[71]
	**Disabling cellular DNA repair process**	Listeriolysin O	*Listeria monocytogenes*	In vitro and epidemiological	Pore formation in intestinal host cells; Prevention of recruitment of repair complex to DNA breaks; listeriosis	[66]
		Secreted effector protein EspF	*Escherichia coli*	In vitro	Down-regulation DNA mismatch repair	[66]
	**Protein metabolism**	Phenol/indol/p-cresol/	Intestinal bacteria	Colonic cells	Increased anion superoxide production and genotoxic effects	[81,82]
		Fecapentanes	*Bacteroides* sp.	In vitro; In vivo	Cytotoxic and mutagenic effects via ROS production; Controversial in vivo effect	[63,83]
		Ammonium	Intestinal bacteria	In vitro	Antiproliferative effect without decrease of cell viability	[84]

## Abbreviations

ACs	Aminocarbolines
AIAs	Aminoimidazoazarenes
ATP	Adenosine triphosphate
B(a)P	Benzo(a) pyrene
CI	Confidence interval
CRC	Colorectal cancer
CYP1A1	Aryl-4 monooxigenase
CYP450	Cytochrome P450
DiMeIQx	2-Amino-3,4,8-trimethylimidazo[4,5-f] quinoxaline
EPIC	European Prospective Investigation into Cancer
HCAs	Heterocyclic amines
IARC	The International Agency for Research on Cancer
LAB	Lactic acid bacteria
MD	Mediterranean Diet
MeIQ	2-Amino-3,4-dimethylimidazo[4,5-f] quinoline
MeIQx	2-Amino-3,8-dimethylimidazo[4,5-f] quinoxaline
MN	Micronucleous assay
MTT	(3-(4,5-Dimethylthiazol-2-yl)-2,5-diphenyltetrazolium bromide)
NAs	Nitrosamines
NOCs	*N*-Nitroso compounds
OECD	Organization for Economic Cooperation and Development
PAHs	Polycyclic aromatic hydrocarbons
PhIP	2-Amino-1-methyl-6-phenylimidazo[4,5-b] pyridine
ROS	Reactive oxygen species
RR	Relative risk
SCE	Sister Chromatid Exchange assay
SCFA	Short chain fatty acids
WD	Western Diet
